# Effect of Heat Treatment on the Property, Structure, and Aggregation of Skim Milk Proteins

**DOI:** 10.3389/fnut.2021.714869

**Published:** 2021-09-17

**Authors:** Hongbo Li, Tingting Zhao, Hongjuan Li, Jinghua Yu

**Affiliations:** ^1^Beijing Advanced Innovation Center for Food Nutrition and Human Health, Beijing Technology and Business University (BTBU), Beijing, China; ^2^State Key Laboratory of Food Nutrition and Safety, Key Laboratory of Food Nutrition and Safety, Ministry of Education, College of Food Engineering and Biotechnology, Tianjin University of Science and Technology, Tianjin, China

**Keywords:** heat treatment, skim milk, property, protein aggregation, 2D-PAGE

## Abstract

To study the mechanism of heat-induced protein aggregates, skim milk was heated at 55, 65, 75, 85, and 95°C for 30 s. Then, the sulfhydryl content, surface hydrophobicity, and secondary structure of heat-treated skim milk were studied. Treating skim milk at different temperatures induced a decrease in sulfhydryl content (75.9% at 95°C) and an increase in surface hydrophobicity (44% at 95°C) with a disrupted secondary structure containing random coil, β-sheet, and β-turn of skim milk proteins. The change in these properties facilitated aggregate formation through disulfide bonds and hydrophobicity interaction. Microstructural observation also showed a higher degree of aggregation when skim milk was heated at 85 and 95°C. The result of two-dimensional polyacrylamide gel electrophoresis demonstrated that the aggregates consisted of a high proportion of κ-casein, β-lactoglobulin, and other whey proteins.

## Introduction

Milk derived from domesticated mammalian animals has a long history of being a part of the human diet. The current milk and milk products for human consumption are mainly from cows, followed by buffaloes, goats, horses, yaks, and camels (1). In the processing of these dairy products, heat treatment is an essential step to reduce the potential risk of survival of pathogenic microorganisms and extend the shelf life of final products. It can also improve the functional properties of some specific dairy products, such as yogurt and cheese (2, 3). Heat treatment has a significant effect on the protein network of dairy product (4). It is well-known that heat-treated milk can form a gel network with better strength and firmness in a shorter time (5). The application of heat leads to different reactions among milk proteins, contributing to the denaturation and/or aggregation of whey proteins and formation of complexes between caseins and whey proteins (6–9).

In dairy proteins, caseins with molecular weight ranging from 19 to 25.2 kDa account for about 80% of total proteins, while whey proteins represent 20%. The main components in whey proteins are β-lactoglobulin (β-LG) and α-lactalbumin (α-LA), representing 50 and 25%, respectively, followed by minor constituents, such as bovine serum albumin (BSA), lactoferrin, and immune globulin (10). In these proteins, caseins are relatively stable at a high temperature, and whey proteins are more susceptible to heat treatment (11). The denaturation of β-LG and α-LA occurs at temperatures above 70–75°C, but the other whey proteins such as BSA and lactoferrin begin to denature at a lower temperature of about 65°C (12, 13). Considerable research studies have been conducted to study the composition of complexes and reactions between proteins (14, 15). Results showed that non-covalent interactions between whey proteins and casein micelles, and the formation of disulfide bond are the most important reactions in the process of heat treatment (16). During the heat treatment of milk, β-LG/κ-casein complexes are formed with the exchange of thiol-disulfide (17). It has been reported that β-LG containing a free sulfhydryl group and two disulfide bonds plays an important role in the formation of β-LG/κ-casein complexes (18, 19).

At present, the effect of heat treatment on milk proteins is mainly focused on the interaction between the proteins, but there are few studies on the properties and structure of total milk proteins. According to the temperature range of whey protein denaturation, 55, 65, 75, 85, and 95°C are selected as the heat treatment temperature on skim milk. The goal of this study is to explore the mechanism of heat-induced protein aggregates after heat treatment on skim milk, and the interaction of different denatured milk proteins.

## Materials and Methods

### Sample Preparation

Fresh cow milk was purchased from a local dairy plant, and 0.02% (w/v) sodium azide was added to prevent bacterial growth. Total protein was determined by the Association of Official Analytical Chemists (20) Method Nos. 991.20 with a conversion factor of 6.38. Cow milk was centrifuged at 2,000 × *g* for 20 min at 4°C to remove the cream, then the skim milk was heated at 55, 65, 75, 85, and 95°C for 30 s in a recirculating tubular heat exchanger made in the laboratory. This heat exchanger contained a tubular coil (1 mm inner diameter) located in a thermostatic water bath and controlled with a constant-flow pump. After heat treatment, the skim milk was rapidly cooled in an ice water bath.

### Sulfhydryl Determination

The sulfhydryl content of the heat-treated samples was determined using the 5,5′-dithiobis (2-nitrobenzoic acid) (DNTB) method. Three hundred microliters of the samples were mixed with 5 ml urea (8 mol L^−1^) and 20 μl DNTB (4 m ml^−1^) (Borunlaite Co., Ltd., Beijing, China) and incubated for 15 min at room temperature. Then, the mixture was immediately measured at 412 nm with a UV-752 UV-vis spectrophotometer (Sunnu Hengping Co., Ltd., Shanghai, China). The mixture without DNTB as a blank control and sulfhydryl content was expressed as micromoles sulfhydryl per milliliter milk.

### Surface Hydrophobicity

The surface hydrophobicity of the heat-treated samples was estimated using the 8-anilino-1-naphthalene sulphonic acid (ANS)-binding fluorimetric assay method. A solution of 8 mmol L^−1^ ANS (Sigma, Shanghai, China) was prepared in a 0.01 mol/L PBS buffer (pH 6.7). The heat-treated samples were also diluted using the PBS buffer to 0.0025, 0.005, 0.01, and 0.02% (w/v), respectively. For each sample, 20 μl ANS was added and equilibrated for 1 h. The fluorescence intensity was measured with a RF-5301 fluorescence spectrophotometer (Shimadu, Shanghai, China), with the excitation wavelength set at 390 nm and the emission wavelength at 470 nm (Ex 390/Em 470). The excitation and emission slits were both set at a bandwidth of 5 nm.

### Fourier Transform Infrared Measurement

The secondary structure of the heat-treated samples was analyzed using a Vector22 FTIR (Bruker, Karlsruhe, Germany) spectrometer with a distributed temperature gradient sensing (DTGS) detector. All spectra were recorded within the range of 4,000–650 cm^−1^ with a 4 cm^−1^ resolution and 32 scans. The measurements were performed in a dry atmosphere at room temperature. In order to reduce the influence of steam absorption in the measurements, dry nitrogen was constantly used.

### Scanning Electron Microscopy

The microstructure of the heat-treated samples was determined using SEM SU1510 (Hitachi, Tokyo, Japan). The heat-treated samples were fixed in 2.5% glutaraldehyde for 3 h at 4°C, and washed three times using a 0.1 mol L^−1^ PBS buffer (pH 7.2). After washing, the samples were dehydrated using 50, 70, 90, and 100% ethanol. Then, ethanol was replaced by isoamyl acetate, and the samples were dried at room temperature. The dried samples were coated with a 4-nm gold layer and observed.

### PAGE Analysis

The aggregates and interactions of proteins in the heat-treated skim milk samples were characterized with the two-dimensional sodium dodecyl sulfate–polyacrylamide gel electrophoresis (2D-PAGE) method. For the first dimension (non-reduced SDS-PAGE), 20-μl samples were loaded and run on 5–15% home-made Tris-HCl gel. The gel strip including aggregates was cut and soaked in a β-mercaptoethanol (β-ME) buffer for 2 h and then placed on top of 5–15% gel for the second dimension (reduced SDS-PAGE). Electrophoresis was carried out in a Tris-glycine electrophoresis buffer. The gel was stained with Coomassie Brilliant Blue R250 and scanned using a Gel Doc XR system (Bio-Rad Laboratories Inc., Hercules, CA, United States). An unstained protein molecular weight marker (14.3–97.2 KDa; ComWin Biotech, Beijing, China) was used in all gels to identify skim milk proteins.

### Statistical Analysis

Data were analyzed with SPSS 14.0 (SPSS Inc, Chicago, IL, United States) and one-way analysis of variance with Duncan's *post-test* was applied. The level of significance was established at *p* < 0.05. All the assays were carried out in triplicate, and data were expressed as mean values ± standard deviation (SD).

## Result and Discussion

### Sulfhydryl Determination

**Figure 1:**
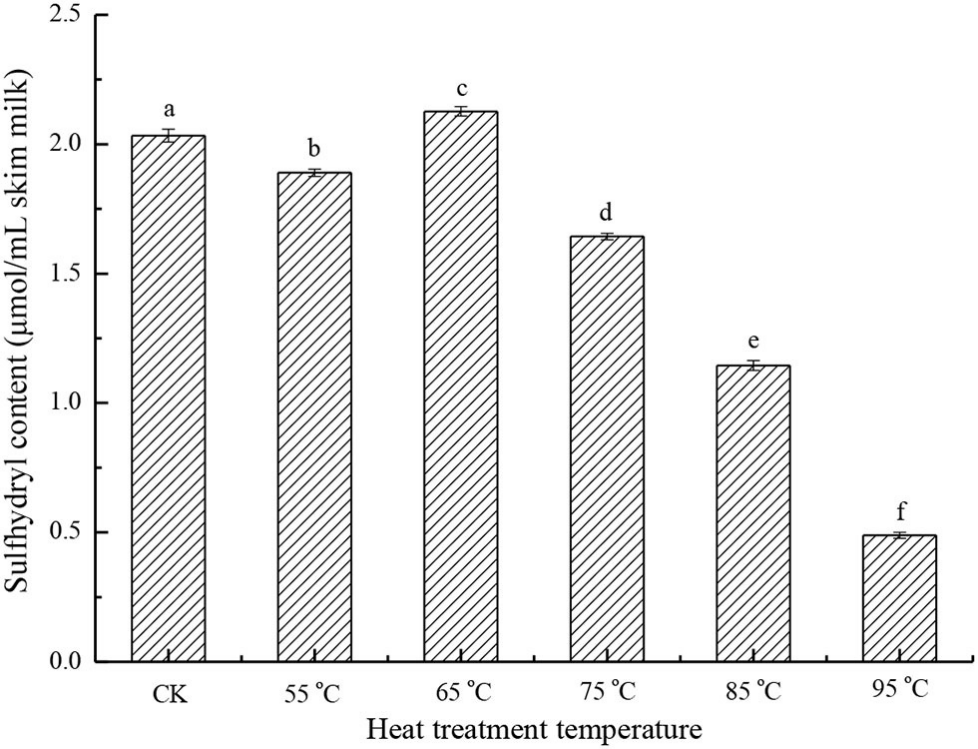
Sulfhydryl content of different heat-treated skim milk. CK, skim milk without heat treatment.

The sulfhydryl content of the heat-treated skim milk samples was studied. As shown in Figure 1, there is no significant change in sulfhydryl content at the lower temperature treatment (55 and 65°C) compared with the sample without heat treatment, but with the increasing of heat treatment temperature, the sulfhydryl content drops significantly (*p* < 0.05). When the treatment temperature reached 95°C, sulfhydryl content was only 24.1% of the control sample. This indicated that heat treatment induced the formation of aggregates through thiol-disulfide exchange. Sulfhydryl is one of the important functional groups in proteins, and it mainly exists in cysteine residues. In milk proteins, sulfhydryl exists in the form of a free thiol group and disulfide bond, and it is important for the maintenance of the native structure of proteins (12). Since heat treatment can cause protein aggregation through thiol-disulfide exchange, and then result in further aggregation *via* hydrophobic association, sulfhydryl content can be used to characterize the aggregation degree of protein samples (17, 21).

In milk proteins, only β-LG has a free thiol group, so the effect of heat treatment on sulfhydryl is mainly focused on β-LG (12). After heat treatment, the free thiol group of β-LG was exposed and involved in the thiol-disulfide exchange reaction, so this free thiol group has the potential to react with other proteins, such as κ-casein and BSA (5, 22). Cho et al. have reported that disulfide bonds and the free thiol group present in β-LG play a very important role in the formation of the β-LG /κ-casein complex by SH/S-S interchange (18). Another possibility to reduce the sulfhydryl content is by the interaction of two β-LG molecules to form a disulfide-bonded dimer (19).

### Surface Hydrophobicity Analysis

**Figure 2:**
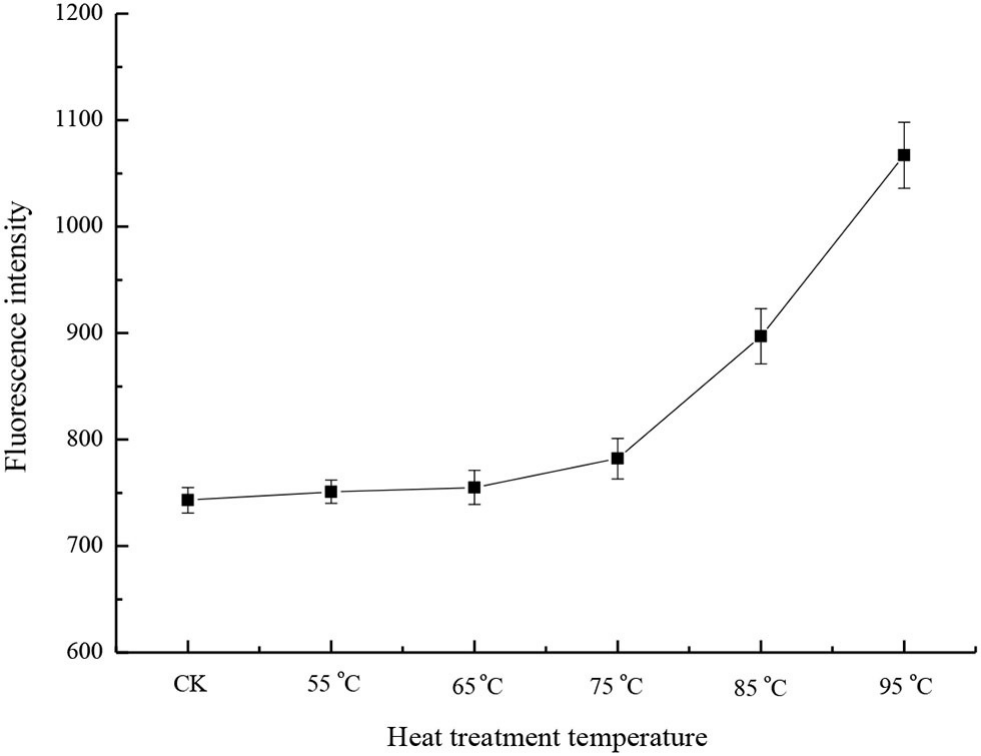
Surface hydrophobicity of different heat-treated skim milk. CK, skim milk without heat treatment.

Hydrophobicity is a property that affects the functionalities of proteins, and is mainly determined by the amino acid composition of proteins. The surface hydrophobicity of the heated samples increased in the heat temperature range of 75 to 95°C (Figure 2). At 95°C, the surface hydrophobicity was 1.44 times that of the control sample. This was consistent with the previous results (23, 24). Hiller and Lorenzen (25) studied the surface hydrophobicity of heat-treated milk proteins such as whey protein isolate, micellar casein, and BSA. The results showed that heat treatment increased the surface hydrophobicity of whey protein isolate and decreased the surface hydrophobicity of BSA, and that the treatment had a little effect on casein. Since BSA accounts for only 1.2% of milk proteins (26), the change in surface hydrophobicity mainly depends on other whey proteins. During heat treatment, β-LG is denatured first, followed by α-LA. When milk was heated at temperatures above 65°C, whey proteins, such as β-LG, α-LA, BSA, and lactoferrin unfolded and exposed previously buried hydrophobic groups, so the surface hydrophobicity was increased at a higher temperature (26). Moreover, the denaturation of whey proteins is reversible at a temperature range of 55 to 65°C, and irreversible at a higher temperature (5, 23).

### FTIR Analysis

**Figure 3:**
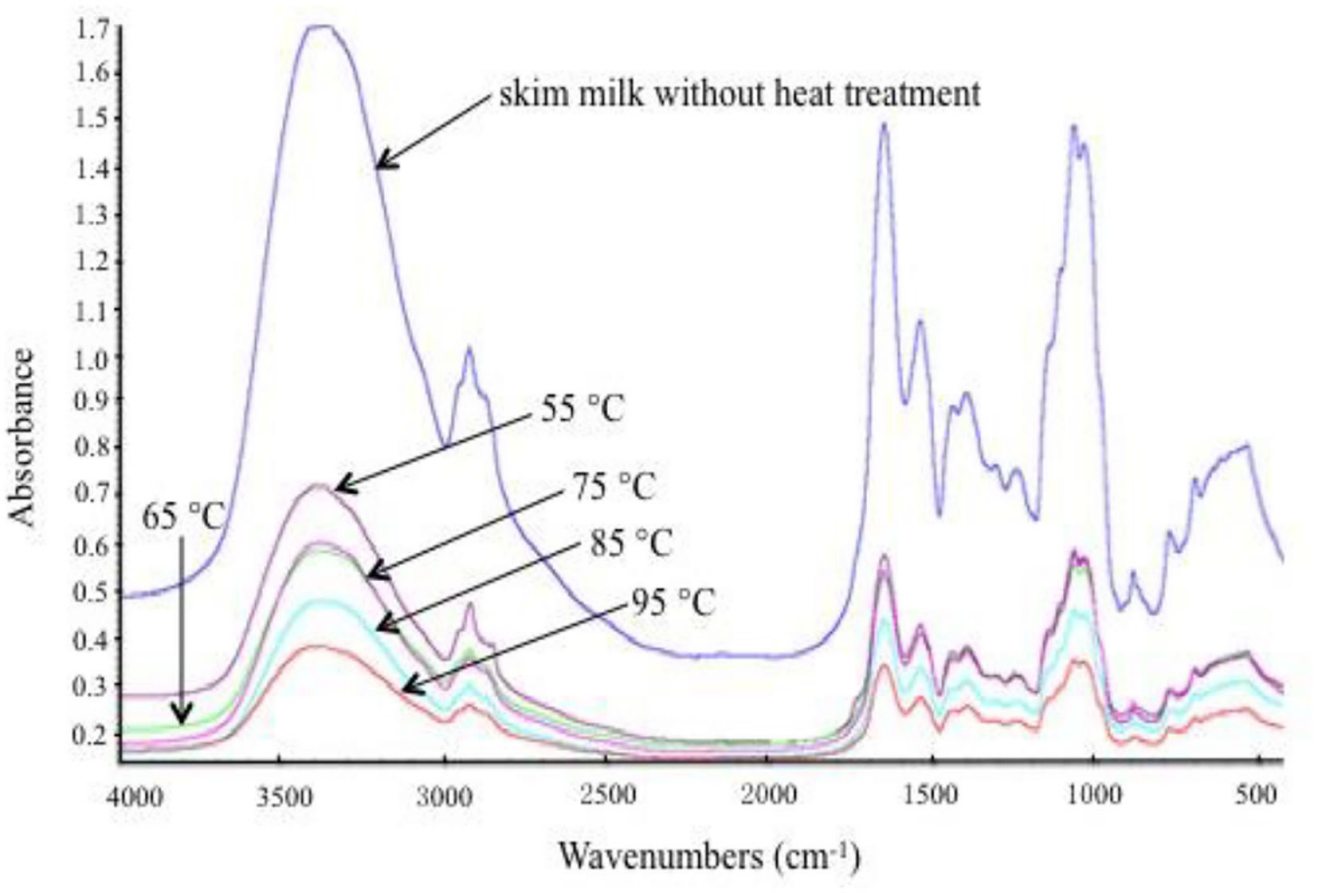
Fourier transform infrared (FTIR) spectra of different heat-treated skim milk.

Fourier transform infrared spectroscopy was selected to obtain the spectrum of skim milk proteins, which illustrated protein conformation. The FTIR spectra of the samples with different heat treatments are shown in Figure 3. Generally, the FTIR spectra of proteins have a strong absorption band in the 1,700–1,600 cm^−1^ region (amide I), which indicates the C = O stretching mode of the peptide chain. In the amide I region, the band that appears at 1,660–1,640 cm^−1^ represents α-helix and random coil, and the band that appears at 1,640–1,620 cm^−1^ represents β-sheet. Figure 3 shows that amide I peak positions have changed slightly in the different samples. Position altering in amide I revealed the change in the secondary structure of milk proteins. In order to obtain further information on secondary structural changes, the OMNIC analysis procedure was used to calculate the proportions of α-helix, β-sheet, β-turn, and random coil in skim milk proteins (Table 1). Random coil was increased, and β-turn was decreased as the heat temperature increased, suggesting the direct conversion of regular β-turn into an irregular random coil with the reduction of the intramolecular hydrogen bond. The reduction of α-helix at 95°C might correspond to the partial unfolding of the α-helix region. The increase in β-sheet might be due to the exposure of hydrophobic regions of milk proteins, which is consistent with the surface hydrophobicity results. Although heat treatment affected the secondary structure of skim milk proteins, this change was not obvious. This might be because caseins, as the main protein in skim milk, mainly existed in casein micelles, and this structure was relatively stable at the above heat treatment temperature (9). Moreover, the subtle change in secondary structure related to the whey proteins. Studies have shown that the content of β-sheet in β-LG is increased when heated above 60°C (12), and that the formation of β-sheet in BSA is irreversible on heating above 70°C (27). The heat treatment of whey proteins caused an abrupt loss in some secondary structures such as disrupted random coil, β-sheet, and β-turn, resulting in the exposure of the free thiol group and, thus, increasing the exposure of inner hydrophobic amino acids (28). This further accelerated the aggregation between milk proteins through disulfide bond and hydrophobic interaction.

**Table 1:** Percentage of secondary structures of skim milk heat-treated at different temperatures.

**Heat treat temperature**	**β-sheet**	**Random coil**	**α-helix**	**β-turn**
Skim milk	30.8	9.4	10.3	49.5
55°C	31.9	9.5	10.1	48.5
65°C	33.3	9.6	10.1	47.0
75°C	32.2	10.7	10.0	47.1
85°C	30.4	12.3	10.3	46.9
95°C	32.0	12.5	9.5	46.0

### Microstructure of Heat-Treated Skim Milk

**Figure 4:**
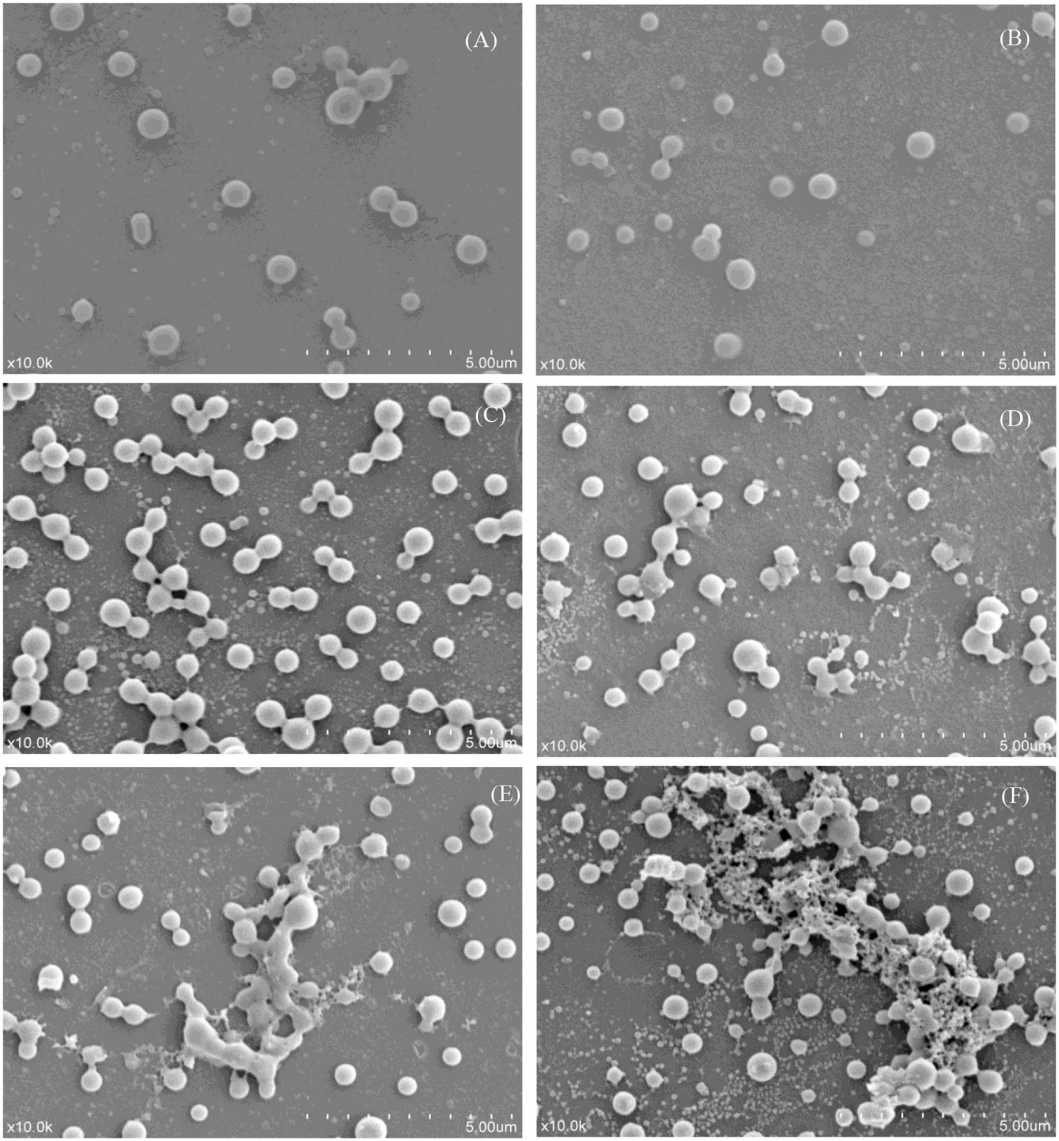
Scanning electron micrographs of different heat-treated skim milk. **(A)** Skim milk without heat treatment; **(B–F)** skim milk heat treated at 55, 65, 75, 85, and 95°C, respectively. All the micrographs were originally taken at a magnification of 10,000 ×. The scale bar represents 5 μm.

Scanning electron microscopy micrographs were obtained from the skim milk samples treated with different temperatures (Figure 4). The SEM images indicated that the morphology of the skim milk proteins heated at 55°C had little change compared with that of the control sample (Figures 4A,B). Caseins (big particles) and whey proteins (small particles) separately dispersed, and no aggregation was formed. The image of samples heated at 65 and 75°C (Figures 4C,D) showed that some proteins aggregated to form casein micellar particles with relatively small whey protein complexes present. The heat treatment of skim milk at 85°C (Figure 4E) showed a higher degree of aggregation between caseins, but also demonstrated the presence of smaller whey protein complexes. The heat temperature of the sample reached 95°C and resulted in a rather loose and reticular structure, with the interaction between casein and whey protein. It was obvious that the aggregation was a large casein cluster aggregated by small whey protein particles (Figure 4F). This aggregation had been demonstrated by disulfide bonds (29) and, to a much lesser extent, by non-covalent bonds (4). Unfortunately, it could not determine which casein and whey protein participated in the aggregation through SEM, so a 2D-PAGE analysis was performed to study the interaction between milk proteins.

### PAGE Analysis

**Figure 5:**
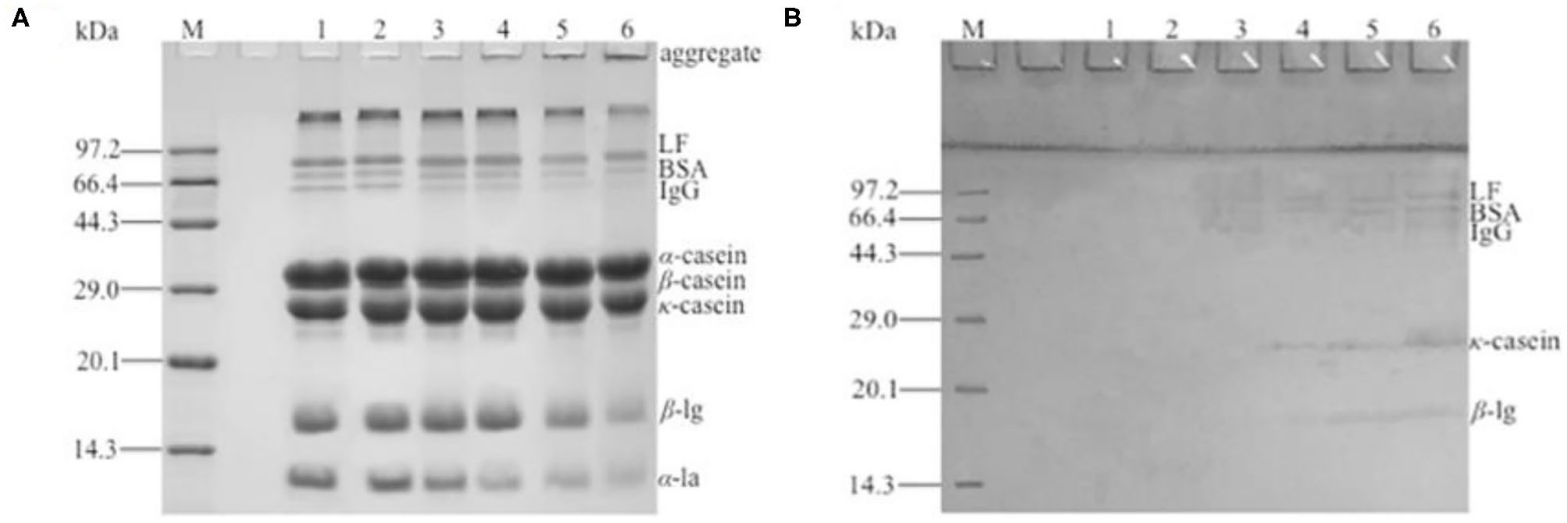
Two-dimensional (2D) sodium dodecyl sulfate–polyacrylamide gel electrophoresis (SDS-PAGE) of different heat-treated skim milk. **(A)** Non-reduced SDS-PAGE of heat-treated samples; **(B)** reduced SDS-PAGE of protein aggregates excised from non-reduced gel. Lane M, protein marker; lane 1, skim milk without heat treatment; lanes 2–6, skim milk heat treatment at 55, 65, 75, 85, and 95°C, respectively.

The heat-treated samples were analyzed by non-reduced SDS-PAGE (Figure 5A) and, after reduction with β-ME, by reduced SDS-PAGE (Figure 5B). As seen in Figure 5A, the intensity of bands corresponding to β-LG and α-LA are markedly reduced with the increase in heat temperature. It can be noted that the lower heat treatment temperatures (55 and 65°C) limited the denaturation and aggregation of β-LG and α-LA, with more than 95% of the above proteins remaining in its native form, similar to the sample without heat treatment, while heat treatment at 95°C promoted the denaturation of most β-LG and α-LA with the rearrangement of the spatial structure. Other minor whey proteins such as BSA, lactoferrin, and immune globulin were very unstable, and the lower intensities of bands that correspond to these three proteins were observed in the samples heat-treated at 65°C. The immune globulin G and BSA bands were essentially absent in the severely heat-treated sample (95°C). Furthermore, large protein aggregates were present in the loading well-indicating molecular weights well-above 200 kDa. The density of the aggregates was enhanced with the increase in heat temperature. By comparison, the SDS-PAGE of reduced samples appeared to be similar (data not shown), indicating that no irreducible covalent bonds formed and that no polypeptide bond was cleaved by the heat treatment (19, 30).

The protein aggregates were excised from the non-reduced gel for the second dimension (Figure 5B). The result showed that a series of bands were present in the SDS-PAGE gel and that the molecular weight was identical to the immune globulin G, BSA, lactoferrin, κ-casein, and β-LG, indicating that the aggregates were formed by the polymerization of the referred proteins. Because immune globulin G, BSA, and lactoferrin were denatured at a lower temperature (13), the aggregates formed at 65°C were mainly these three proteins. With the increase in heat treatment temperature, κ-casein and β-LG participated in the polymerization. A polymerization experiment of α-LA, β-LG, κ-casein, and BSA by dissolving two of them in simulated milk ultrafiltrate was done (data not shown). The result showed that β-LG could react with other β-LG or κ-casein and accelerate the denaturation and aggregation of α-LA. BSA responded to the heat treatment similarly to β-LG, except that BSA reacted at a lower temperature. This result further demonstrated that the heat-induced aggregates mainly composed by β-LG, κ-casein and BSA. This was similar with the previous reports (5, 19, 31). It was generally considered that β-LG could react with other β-LG or κ-casein through disulfide bonds (17, 18). The result of sulfhydryl content showed that the free thiol group was used to form disulfide bond with other milk protein, so the sulfhydryl content dropped significantly with the increase in heat treatment temperature. In general, reactivity of free thiol group is dependent on the protein unfolding degree, as well as disulfide interchange occurring at high temperature (29). In the SDS-PAGE, monomer structure of κ-casein and β-LG were observed after aggregates were reduced by β-ME. This indicated that the new disulfide bond existed in the aggregates, and this result was consistent with the decrease in sulfhydryl content. However, α-LA was not observed in the SDS-PAGE, and this might be because of the intensity of aggregates containing α-LA was too low to observe. Although α_s2_-casein, which had two Cys residues, did not react with β-LG, it was present as a dimer with intermolecular disulfide bonds (32). This was because α_s2_-casein was not located at the surface of the micelle, and the disulfide bonds were not accessible to the denaturing or denatured β-LG (30). The aggregates of the other two caseins, α_s1_-casein and β-casein (12), were formed by hydrophobic interaction, so α_s1_-casein and β-casein were absent from the SDS-PAGE gel.

## Conclusion

In this study, the mechanism of heat-induced milk protein aggregates was investigated in detail. The result showed that heat treatment decreased sulfhydryl content, increased surface hydrophobicity, and disrupted the secondary structure of milk proteins. The change in these properties accelerated the denaturation of milk proteins and the formation of aggregates linked by disulfide bonds and hydrophobicity interaction. Moreover, the aggregates were formed at high heat temperature mainly composed of κ-casein, β-LG, and other whey proteins.

## Data Availability Statement

The original contributions presented in the study are included in the article/Supplementary Material, further inquiries can be directed to the corresponding author.

## Author Contributions

This study was conceived, designed, and written by HongbL. TZ performed the experiments and analyzed the data. HongjL improved the manuscript. JY provided experimental technical support. All authors contributed to the article and approved the submitted version.

## Funding

This study was financially supported by the National Key Research and Development Program of China (Grant No. 2017YFE0131800), and Tianjin Municipal Education Commission (Grant No. 2018KJ092).

## Conflict of Interest

The authors declare that the research was conducted in the absence of any commercial or financial relationships that could be construed as a potential conflict of interest.

## Publisher's Note

All claims expressed in this article are solely those of the authors and do not necessarily represent those of their affiliated organizations, or those of the publisher, the editors and the reviewers. Any product that may be evaluated in this article, or claim that may be made by its manufacturer, is not guaranteed or endorsed by the publisher.
