# Supplementary material for: Effect of Heat Treatment on the Property, Structure, and Aggregation of Skim Milk Proteins
Source: Front Nutr. 2021 Sep 17;8:714869. doi: 10.3389/fnut.2021.714869 (PMC8485980; doi:10.3389/fnut.2021.714869)
Supplement: Supplementary file 1 [file Data_Sheet_1.PDF]

Supplementary materials

Reduced SDS-PAGE

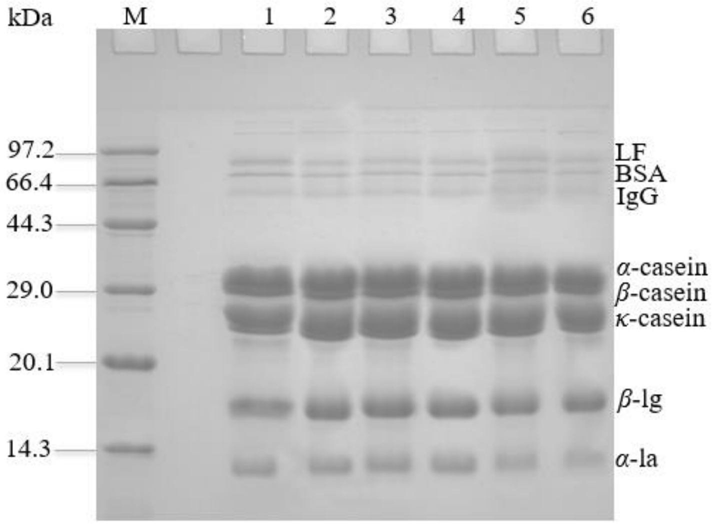

Fig.1 Electropherograms of different heat treated skim milk. Lane M, protein marker; lane 1, skim milk without heat treatment; lane 2-6, skim milk heat treatment at 55 °C, 65 °C, 75 °C, 85 °C and 95 °C respectively.

The polymerization experiment of same milk proteins

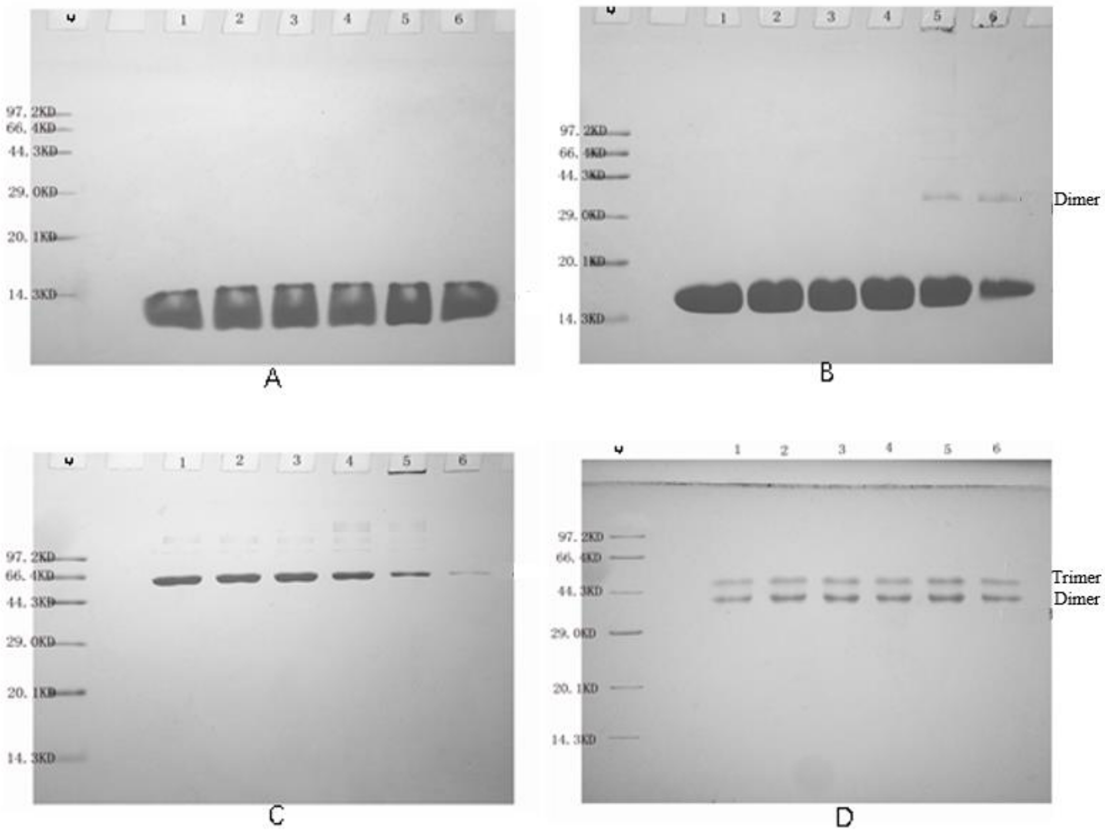

Fig.2 Electropherograms of different heat treated α-LA (A), β-LG (B), BSA (C) and κ-casein (D). Lane 1, protein without heat treatment; lane 2-6, protein heated at 55 °C, 65 °C, 75 °C, 85 °C and 95 °C respectively.

The polymerization experiment of two milk proteins

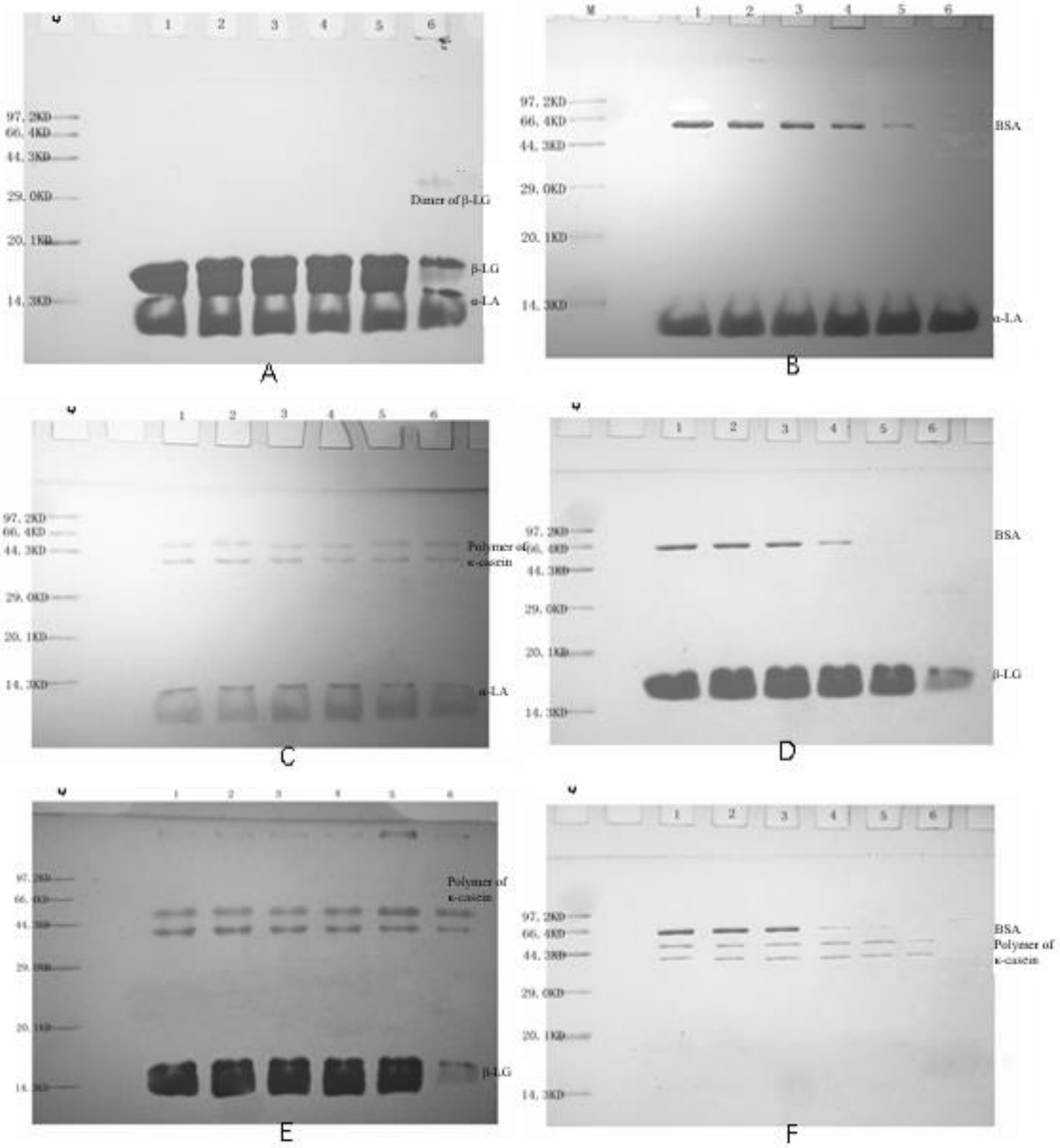

Fig.3 Electropherograms of interaction between α-LA + β-LG (A), α-LA + BSA (B), α-LA + κ-casein (C), β-LG + BSA (D), β-LG + κ-casein (E), BSA + κ-casein (F) after heat treatment. Lane 1, protein without heat treatment; lane 2-6, protein heated at 55 °C, 65 °C, 75 °C, 85 °C and 95 °C respectively.
